# Molecular Insights into the Binding and Conformational Changes of Hepcidin25 Blood Peptide with 4-Aminoantipyrine and Their Sorption Mechanism by Carboxylic-Functionalized Multiwalled Carbon Nanotubes: A Comprehensive Spectral Analysis and Molecular Dynamics Simulation Study

**DOI:** 10.1021/acsomega.4c04515

**Published:** 2024-08-06

**Authors:** Reza Rasoolzadeh, Leonardo Baptista, Fahimeh Sadat Vajedi, Vahid Nikoofard

**Affiliations:** † Department of Inorganic Chemistry, Institute of Chemistry, Fluminense Federal University, Niterói, Rio de Janeiro 24020-140, Brazil; ‡ Department of Chemistry and Environmental, Faculty of Technology, Rio de Janeiro State University, Resende, Rio de Janeiro 27537-000, Brazil; § Department of Chemistry, Institute of Chemistry, Rio de Janeiro State University, Rio de Janeiro, Rio de Janeiro 20550-900, Brazil; ∥ Department of Mathematics, Physics and Computation, Faculty of Technology, Rio de Janeiro State University, Resende, Rio de Janeiro 27537-000, Brazil

## Abstract

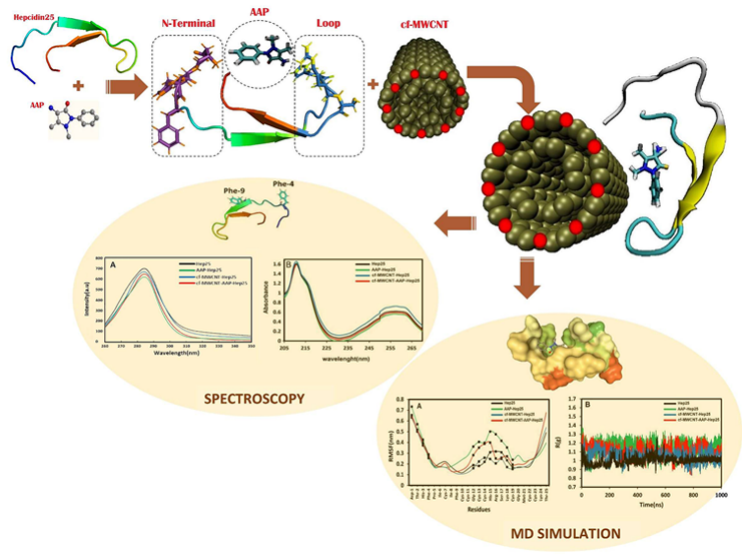

In this work, the main purpose is to analyze and understand the mechanism and thermodynamic interactions of carboxylic acid-functionalized multiwalled carbon nanotubes (cf-MWCNTs) and 4-aminoantipyrine (AAP) with human hepcidine25 (Hep25) using multispectroscopic and molecular docking modeling methods, binding free energy calculations, and molecular dynamics (MD) simulations under physiological conditions. AAP belongs to a class of persistent environmental contaminants, and its residue is a potential hazard to human health, exhibiting a high binding affinity with blood peptides. Hepcidin is a 25-residue peptide hormone with four disulfide bonds that regulates the iron balance in vertebrates and contributes to host immunity as a cysteine-rich antimicrobial peptide. Due to their diverse properties and pollutant absorption capabilities, CNTs demonstrate important biological effects in biological applications, particularly in the noncovalent interactions with blood peptides. A comprehensive molecular dynamics simulation integrated with molecular docking methodologies was employed to explore the binding free energy between AAP and Hep25, identify binding sites, elucidate thermodynamic characteristics, and evaluate the binding forces governing their interaction. The investigation delved into elucidating the precise binding site of AAP within the Hep25 protein and thoroughly analyzed the impact of AAP on the microenvironment and conformational dynamics of Hep25. The circular dichroism (CD) experimental results highlight a reduction in β-sheet composition following the introduction of AAP and cf-MWCNT. In addition, outcomes from fluorescence spectroscopy demonstrate that both cf-MWCNT and AAP significantly attenuated Hep-25 fluorescence via a static quenching mechanism. According to the MD simulations, the presence of AAP induces changes in the secondary structure of Hep25 and enhances its hydrophobicity. Additionally, our findings demonstrated that alongside the alteration in protein structure and functionality induced by contaminants, cf-MWCNTs possess the capability to mitigate the contaminant-induced effects on Hep25 activity while preserving the overarching structural integrity of Hep25. Based on the distance and RDF data, we found that during the simulation the presence of the cf-MWCNT causes the AAP to move away from the Hep25, and as a result fewer and weaker interactions of the AAP with the Hep25 will be observed. Likewise, free energy calculations indicate that the binding of Hep25 to AAP and cf-MWCNT involves electrostatic, π-cationic, and π–π stacking interactions. The research findings offer invaluable insights into the intricate influence of pollutants and carbon nanotubes on protein functionality within the circulatory system and their toxicity in vivo for prospective investigations.

## Introduction

Hepcidin, a 25-residue iron-regulatory peptide, is made up of eight cysteine residues linked together by four disulfide bonds, giving rise to a molecule with a hairpin-like structure and two arms joined by disulfide bridges in a ladder-like arrangement. Besides the predominant form containing 25 amino acids, 20 and 22 residues of hepcidin have also been found in human urine, the only difference being their N-terminus amino acids. In both hepcidin25 (Hep25) and hepcidin20 (Hep20), structures with a disulfide-paired core of Cys residues and hydrogen bonds between their antiparallel strands confirm a distorted β-sheet model with a hairpin loop. The primary cellular source that produces hepcidin is the hepatocyte. Still, recent studies have shown that bacteria-activated neutrophils and macrophages can produce this substance at a lower level than that in hepatocytes.^1^ Hepcidin pre-propeptide with 84 amino acids, comprising a 24-residue peptide at the N-terminus, a 35-residue pro-region, and a mature peptide with 20 or 25 residues at the C-terminus, is the predominant form in human urine.^2^ The peptide regulates iron export from storage cells, including macrophages and hepatocytes, by binding to ferroportin, the iron exporter protein. This results in the internalization and degradation of the iron transporter. In reality, iron overload can trigger the expression of hepcidin, leading to increased erythropoiesis and hypoxia and regulating anemia levels through hepatocytes.

Proteins are paramount bioactive entities within biological systems, underscoring their pivotal role in life processes; any fluctuation in protein concentrations, whether they decrease or increase, can serve as a fundamental biomarker for clinical diagnostics and health appraisal. Various factors can influence proteins’ normal physiological functions and conformational changes. In addition to endogenous physiological conditions (such as pH and temperature), exogenous environmental pollutants can also impact protein conformation and function, harming human health by interfering with diverse biomolecules.^3^ Recent studies have extensively investigated the interaction of small molecules, drugs, and pollutants with proteins, especially elucidating the structural aspects of their binding to evaluate the structure–function relationship and efficacy.^4,5^ Pollution toxicity studies depend on their interactions with biomolecules. The study conducted by Ju et al. delved into the molecular intricacies of poly(vinyl chloride) microplastics (PVC MPs) in their interaction with bovine serum albumin, focusing on the elucidation of the binding affinity. Through a series of comprehensive analyses, the research unveiled significant alterations in the microenvironment and secondary structure of BSA upon exposure to PVC MPs.^6^ Wei and colleagues^7^ meticulously explored the impact of hydroxylated polybrominated diphenyl ethers (OH-PBDEs) as hazardous environmental contaminants on the thyroid transporter (TTR). Computational simulations revealed that OH-BDE induced notable modifications within the internal milieu of TTR, consequently precipitating alterations in its secondary structural attributes. These observations underscore the profound influence of OH-PBDEs on TTR’s structural integrity and functionality, shedding light on potential mechanisms underlying their toxicological effects at a molecular level.

The chemical diversity of pollutant molecular structures not only influences the conformation of biomolecules but also contributes to binding modes and binding free energies between pollutants and biomolecules. Moreover, combining pollutants with proteins can lead to toxic effects and alteration of the protein’s secondary structure. Previous reports indicate that organic pollutants primarily bind to proteins such as serum albumin, estrogen receptors (ERs), androgen receptors (ARs), aryl hydrocarbon receptors (AHRs), blood proteins, and thyroid transporters (TTRs).^8^ 4-Aminoantipyrine (AAP), possessing an aromatic structure and belonging to the pyrazolone group, exhibits analgesic, antipyretic, and anti-inflammatory properties. Although uncommon as an analgesic due to potential side effects like agranulocytosis, AAP is mainly employed as an intermediate for synthesizing pharmaceuticals with improved biological properties, such as antipyretics and analgesics.^9,10^ It has also been reported that 4-aminoantipyrine is toxic even at low levels of exposure when injected into laboratory animals.^11^ Furthermore, AAP can decrease concentrations of 13,14-dihydro-15-keto prostaglandin F2-α^12^ and reduce blood flow^13^ after infusion into the body. AAP demonstrates a high affinity for heme, as evidenced by its ability to form enduring complexes.^14^ When interacting with circulating proteins, AAP exerts a significant destabilizing effect, particularly impacting their structural conformation in biological systems.^15−17^

Regarding the adverse effects of AAP on proteins, currently there are no data on the binding of AAP and its metabolites to Hep25 proteins at the molecular and experimental levels. The interaction mechanisms of AAP with bovine hemoglobin (BHb) were investigated by Teng et al. through a comprehensive array of experimental techniques including fluorescence, UV–vis, and CD spectroscopy. The study showed that AAP profoundly impacts the hydrogen bonding networks inherent within the polypeptide chain of BHb. These perturbations inevitably disrupt the structural integrity and normal functionality of BHb, posing a tangible risk of toxicity within the biological system.^16,18^

The heightened apprehensions surrounding the harmful impacts of AAP on human health and environmental ecosystems have catalyzed a surge in research endeavors on the efficacious mitigation of these compounds. A safe and effective disposal method must be identified to manage pollutants effectively and safely without adverse environmental impacts. Therefore, using new and advanced materials is crucial for detecting small quantities of contaminants in the human body, compensating for the lack of treatment technologies and analytical methods. Recent research has mainly focused on nanostructures such as carbon nanotubes (CNTs) as effective adsorbents and catalysts for eliminating harmful and toxic contaminants from the environment. Hu et al. conducted an in-depth investigation into the sorption mechanism of polycyclic aromatic hydrocarbons (PAHs) by CNTs utilizing DFT calculations, comprehensive atomistic MD simulations, and rigorous binding free energy calculations.^19^ Results revealed a nuanced interplay in which contaminants exhibited dynamic motion during MD simulations while maintaining consistent π–π stacking interactions with the CNT surfaces. Moreover, the study posits CNTs as promising candidates for the sorption of hydrophobic contaminants, shedding light on their potential application in environmental remediation strategies.

In addition, the combination of a high surface area and a layered and hollow structure in carbon nanotubes makes them suitable for supporting drug molecules, proteins, and gene transfer through chemical and physical processes.^20^ CNTs have demonstrated notable utility in the realm of biomolecule separation, particularly in the isolation of peptides and proteins. Through both covalent and noncovalent functionalization, CNTs serve as discerning agents, effectively sieving and capturing specific molecules akin to sentinels. The efficacy of this selective process hinges significantly on the dimensions and characteristics inherent to the nanostructure. Furthermore, two different methods, chemical and physical modifications, have been proposed for the preparation of soluble CNTs; both reduce the toxicity of CNTs to a large extent and expand their safe use in nanobiotechnology and nanomedicine. Extensive research endeavors have been dedicated to elucidating the biological ramifications of carbon nanotubes. Among the focal points of these investigations are the intricate interactions between proteins and CNTs, often referred to as the nanoparticle–protein corona, which is recognized as a pivotal determinant in shaping the biological effects of CNTs.^21,22^ As reported previously, the interactions of multiwalled carbon nanotubes (MWCNTs) with biological macromolecules like nucleic acids and proteins, in addition to the structural changes of proteins, play a significant role in determining their uptake by cells and modulating their toxicity.^23,24^

Furthermore, interactions between specific proteins and CNTs have demonstrated potential for augmenting biocompatibility and rendering protein-modified nanotubes nontoxic or less toxic than their pristine counterparts.^25^ The affinity of proteins for nanoparticle surfaces is contingent upon several factors, including surface characteristics, size, curvature, composition, and the specific preparation methodology employed.^26^ Understanding the interaction processes for safety purposes and developing and optimizing new pollutant–protein–CNT modification technologies is necessary. Carbon nanotubes are loaded with a protein through two possible interactions: attachment and encapsulation.^27^ Additionally, molecular interactions between proteins and carbon nanotubes through covalent or noncovalent forces help increase the biocompatibility of nanotubes while causing a stable thermodynamic structure after absorption. Therefore, the interaction of nanotubes, used as pollutant absorbers, with proteins reduces the secondary structural and functional changes of proteins that occur as a result of the interaction with pollutants.^28^ On the other hand, reducing the toxic effect of pollutants on proteins and creating an isolated environment protects them from destruction and interaction with healthy cells.

A comprehensive examination of the intricate interplay between pollutants and proteins is imperative to elucidate the profound impact of pollutant–protein binding on protein structure, function, and response mechanisms because the existing comprehension of these pollutant-biomolecule interactions is complex and requires an intensified research focus.^29^ Although there are various applications of CNTs in detecting and removing organic pollutants, the precise mechanism of how AAP is adsorbed on CNT–protein surfaces is not well understood. Martins et al. modified oxidized multiwalled carbon nanotubes by functionalizing them with bovine plasma (BP@MWCNT) to assess their efficacy in the removal of copper from water sources. Notably, the functionalized material exhibited no discernible acute toxic effects on *Daphnia similis*.^30^ Given the abundance of existing environmental pollutants, experimental tests on the adsorption of numerous pollutants to proteins become impractical due to the workload and high cost. The ecological risk and pollution inhibition caused by organic chemicals may be assessed by expanding the adopted computational methodology to simulate and estimate the pollutants’ adsorption on proteins. Computational simulations are more efficient methods for investigating absorption processes due to the limitations of laboratory methods for studying the interactions between different molecules and pollutants. Molecular dynamics (MD) simulations offer a robust methodology for investigating intricate associations between macromolecular structures and functionalities, and the current simulation durations closely approximate biologically pertinent timeframes. They become very popular when large systems or long simulations are needed to display the systems at different levels of detail. Lui et al. investigated the interaction of AAP with bovine serum albumin by spectroscopic and molecular docking.^16^ We previously performed an MD method to study the effect of a single-walled carbon nanotube (SWCNT) on human hepcidin.^31^ Using the semiempirical and Monte Carlo methods, we also investigated interaction energies of carbon and BN nanotubes with human hepcidin peptides.^32^ According to our knowledge, until now, there has been no research report on binding and structural changes related to the effect of pollutants on CNT–biomolecule complexes using multiple spectroscopic techniques and molecular dynamics simulation.

Given the pivotal role of Hep25 in cardiovascular physiology, it would be intriguing to investigate how AAP influences the functionality and dynamics of Hep25 within the circulatory system. The present work performs an accurate structural and conformational examination of binding of the AAP pollutant to Hep25 using several biophysical methods such as fluorescence, UV, and CD spectroscopy. Furthermore, an MD simulation combined with molecular docking was implemented to investigate the binding free energy between AAP and Hep25, binding sites, thermodynamic parameters, and binding force for their interaction. The specific binding site of AAP on Hep25 and the effect of AAP on the microenvironment and conformation of Hep25 were investigated in detail. We also revealed that, in addition to the contaminant effect on protein structure and activity, carboxylic acid-functionalized multiwalled carbon nanotubes (cf-MWCNTs) can reduce the contaminant activity on Hep25 without significantly impacting the overall structure of the Hep25. Thus, this study aimed to understand the mechanism of interaction of AAP with Hep25 and cf-MWCNT-Hep25 through experimental and MD simulation results. This research aims to elucidate the intricate binding interactions between AAP and Hep25 and cf-MWCNT-Hep25 complexes by utilizing a comprehensive approach involving experimental analyses and molecular dynamics simulations.

These findings can help to uncover the effect of pollutants and CNTs on protein function during transport in blood and the toxicity in vivo for future studies.

## 2 Materials and Methods

### 2.1 Materials

Human hepcidin25 peptide was purchased from Abcam (USA). Tween 20 (99%) was provided by Merck (Germany). HEPES buffer (99.5%) was purchased from Sigma. 4-Aminoantipyrine (99%) was obtained from Merck. Carboxylic acid-functionalized MWCNTs (cf-MWCNTs) (95%) (avg. diameter × length of 9.5 nm × 1.5 μm) were purchased from Sigma. All other chemicals used in this study, such as ethylenediaminetetraacetic acid (EDTA) (99.99%), Tween 20, and NaCl, were of analytical reagent grade (Merck or Sigma) and used as received. Double-distilled water and ethanol were used as the solvents.

### 2.2 Experimental Method

The Hep25 solution was prepared in 20 mL of buffer (30 mM HEPES buffer, 150 mM sodium chloride, 0.001% Tween 20, and 2 mM EDTA), with a pH of 6.7–7 to form a 2.5 × 10^–5^ M solution, and it was stored at 4 °C prior to use. AAP was dissolved thoroughly in deionized water and stirred vigorously to form a 1.0 × 10^–4^ mol L^–1^ homogeneous solution. The required amount of cf-MWCNT (2.73 wt %) was dispersed in ultrapure water by ultrasonication for 1 h before the experiment.

### 2.3 Fluorescence Measurements

All fluorescence spectra were recorded on a Cary eclipse fluorescence spectrophotometer (Varian, Australia) using a 10 mm quartz cell. The photomultiplier tube (PMT) voltage was set to 700 V, and the excitation and emission slit widths were fixed at 5 nm. The emission wavelength was obtained at 282 nm upon excitation at 253 nm. The scan speed was 600 nm min^–1^. Intrinsic fluorescence of 2.5 × 10^–5^ M Hep25 in buffer, at pH 7.0, was measured in the absence and presence of cf-MWCNT and AAP. Hep25 solution absorption was subtracted from the conjugate absorption spectra in all measurements.

### 2.4 UV–visible Absorption Measurements

The UV–visible spectra of as-prepared samples were recorded using a Nanodrop 2000 spectrophotometer (Thermo Scientific Nanodrop) equipped with a 10 mm quartz cell in the wavelength range of 190–400 nm at room temperature. The peptide concentration used for this experiment was 2.5 × 10^–5^ M. To smooth the UV spectra, all of the spectra containing cf-MWCNT-Hep25, cf-MWCNT-AAP-Hep25, and AAP-Hep25 solutions were subtracted from the spectra of the Hep solution.

### 2.5 Far-UV Circular Dichroism Measurements

The Circular dichroism (CD) measurements were recorded on a J-810 CD spectropolarimeter (Jasco, Tokyo, Japan) in the 190–250 nm wavelength range using a 1 mm quartz cell. The scanning speed was set at 200 nm min^–1^. The results are presented as molar ellipticity regarding ellipsoidal size and the peptide’s average amino acid residue weight (MRW). Data smoothing was performed by Jasco J-810 software, which includes a fast Fourier transform noise reduction routine.

### 2.6 Molecular Docking

In this study, Auto Dock 4.2^33^ was used to search for conformation models and establish 3D structures of the compounds. Additionally, the protein was prepared before the relevant dockings by removing water molecules and other excess ligands, securing all hydrogen atoms, and adding charges. The binding modes of AAP to Hep25 were then determined considering the protein’s binding site and setting the GridBox size to 90 × 90 × 90 Å^3^. With VMD software, we constructed a single-walled carbon nanotube possessing chirality (9, 9). The docking process, consisting of 20 different independent conformations, was performed using Lamarckian genetic algorithms (GA) for each complex, and eventually the lowest binding energy was chosen for docking analysis.

**Figure 1:**
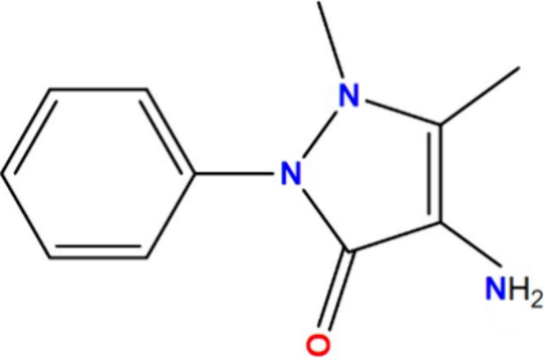
Structure of 4-aminoantipyrine (AAP).

4-Aminoantipyrine, also known as ampyrone, is an amine derivative characterized by multiple functional groups including a carbonyl group. Its pentacyclic structure incorporates two nitrogen atoms, which contribute to the overall aromaticity of the compound (Figure 1). As a result, 4-aminoantipyrine has been extensively used across various domains within the realm of chemistry. The structure of AAP used in the molecular simulations was previously optimized at the B3LYP/cc- pVTZ level^34^ in the Gaussian 09 package.^35^ The initial coordinates of Hep25 were taken from the protein database (PDB ID 1M4F for Hep25).

### 2.7 Molecular Dynamics Simulation

**Figure 2:**
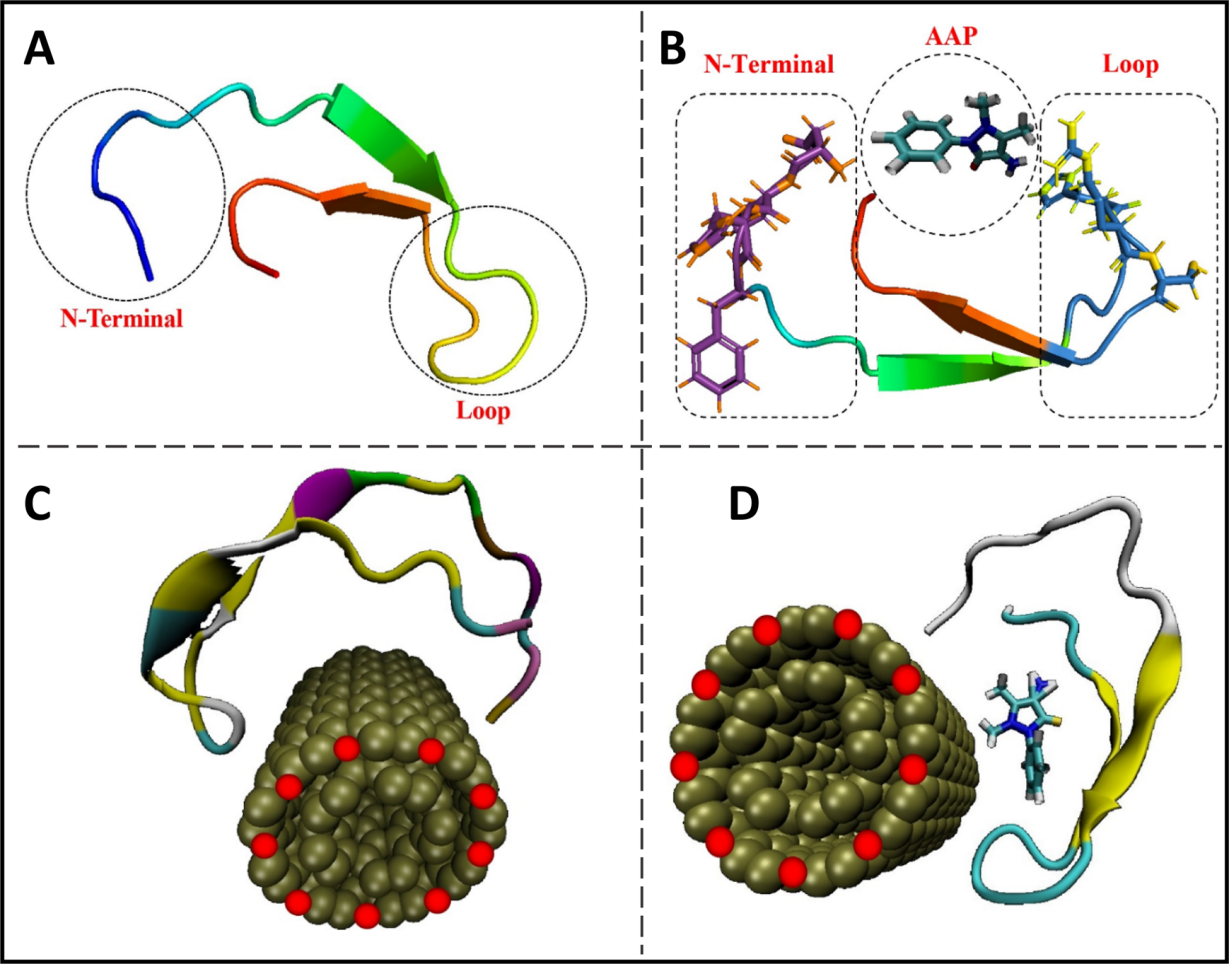
(A) Structure of the native state of Hep25. Snapshot of complexes after 600 ns of MD for (B) AAP-Hep25, (C) cf-MWCNT-Hep25, and (D) cf-MWCNT-AAP-Hep25 complexes, respectively.

The behavior of the dynamic adsorption of the selected pollutant on the protein surface and the AAP-Hep25, cf-MWCNT-Hep25, and cf-MWCNT-AAP-Hep25 complexes was investigated in an aqueous environment using molecular dynamics simulations. The GROMACS simulation software package (version 5.1.2) with the GROMOS 54A7 force field was chosen in all simulations to determine topology parameters and analyze adsorption systems.^36,37^ Using an online PRODRG server, we created the topology file of the small molecule AAP. In this research, a cf-MWCNT was utilized (containing the armchair with an inner (5, 5) tube and an outer (9, 9) tube) with terminal carboxylation. The simulation involved characterizing carbon atoms as neutral Lennard-Jones entities utilizing atomistic parameters specific to aromatic carbon constituents. The systems were simulated in pure water to obtain comparative results. Hep25 and the AAP-Hep25, cf-MWCNT-Hep25, and cf-MWCNT-AAP-Hep25 complexes were located in the center of a cubic simulation box (with dimensions 90 × 90 × 90 Å^3^) and combined with solvents in the systems using a simple point charge (SPC) water model.^38^ MD simulations of the aqueous protein and complexes were carried out close to the desired quantities (1 bar and 300 K) using the Berendsen algorithm with coupling coefficients of τ_*T*_ = 0.1 ps and τ_*p*_ = 0.1 ps, respectively.^39^ The energy minimization was conducted for the whole system for 1000 ps in the NVT and NPT ensembles.^40^ The charge neutralization of the overall simulation system was done by adding sufficient quantities of sodium (Na^+^) and chloride (Cl^–^) ions to the simulation boxes. LINCS was applied to limit all covalent bonds involving hydrogen atoms.^41^ A short-range spherical cutoff of 1 nm was employed for all nonbonded interactions. The particle mesh Ewald (PME) method was adopted to compute the long-range electrostatic interactions.^42^ Simulations were conducted using periodic boundary conditions and the single-point charge (SPC) water model with a typical liquid density (55.32 mol L^–1^). A 1000 ns trajectory was simulated as a final step to reach a constant oscillation of the root-mean-square deviation and the system’s total energy. The reference structure of the Hep25 and the representative snapshots of the three distinct simulation systems, consisting of AAP-Hep25, cf-MWCNT-Hep25, and cf-MWCNT-AAP-Hep25, are shown in Figure 2.

### 2.8 Binding Free Energy Calculations

Utilizing MD simulation outcomes, we conducted a molecular mechanics–Poisson–Boltzmann surface area (MM-PBSA) analysis to scrutinize complex interactions and evaluate the binding free energies (Δ*G*_binding_) among AAP, Hep25, and cf-MWCNT.^43,44^ The g_MMPBSA tool was employed to compute the binding free energy.^45^Equation 1 outlines the computation of the binding energy.
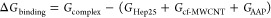
1

*G*_complex_ represents the overall free energy of the complexes, while Δ*G*_Hep25_, Δ*G*_AAP_, and Δ*G*_cf-MWCNT_ denote the individual free energies of Hep25, AAP, and cf-MWCNT in the solvent, respectively. Each complex’s contributory interacting free energies comprise three distinct energetic terms, collectively influencing the total binding free energy (eq 2).
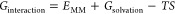
2

In this context, *E*_MM_ represents the molecular mechanics energy term, *G*_solvation_ denotes the free energy of solvation, and *T* and *S* are the absolute temperature and molecule entropy, respectively. It is worth noting that, in alignment with the typical approach in many computational studies, the peptides’ entropy contribution (*TS*) was omitted from consideration. This study’s primary objective was to ascertain each system’s binding free energies.

Δ*E*_MM_ was calculated with the following equation (eq 3):
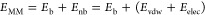
3where *E*_b_ is the bonding interactions and *E*_nb_ is the nonbonding interactions, which are calculated as the summation of electrostatic (elec) and van der Waals (vdw) interaction energies. Δ*E*_b_ is usually taken to be zero. Electrostatic interactions were computed using the Coulomb potential function, while van der Waals interactions were calculated via the Lennard-Jones (LJ) potential function.

The polar binding energy (Δ*G*_polar-binding_) and nonpolar binding energy (Δ*G*_nonpolar-binding_) were determined using the following equations (eqs 4 and 5):
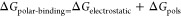
4
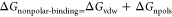
5

The solvation-free energy (*G*_solvation_) was determined by multiplying the electrostatic (*G*_polar_) and apolar (*G*_nonpolar_) solvation free energies.
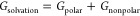
6

*G*_polar_ was computed by employing the Poisson–Boltzmann (PB) equation, and *G*_nonpolar_ was estimated by utilizing the solvent-accessible surface area (SASA) as follows:
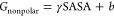
7with empirical parameters set to γ = 0.0054 kcal mol^–1^ Å^–2^ and *b* = 0.92 kcal mol^–1^.^46,47^

The interactions between AAP-Hep25, cf-MWCNT-Hep25, and cf-MWCNT-AAP-Hep25 complexes were assessed based on van der Waals (*E*_vdw_) interaction energies and solvent-accessible surface area (SASA) energies.

## 3 Results and Discussion

### 3.1 Spectroscopy Section

**Figure 3:**
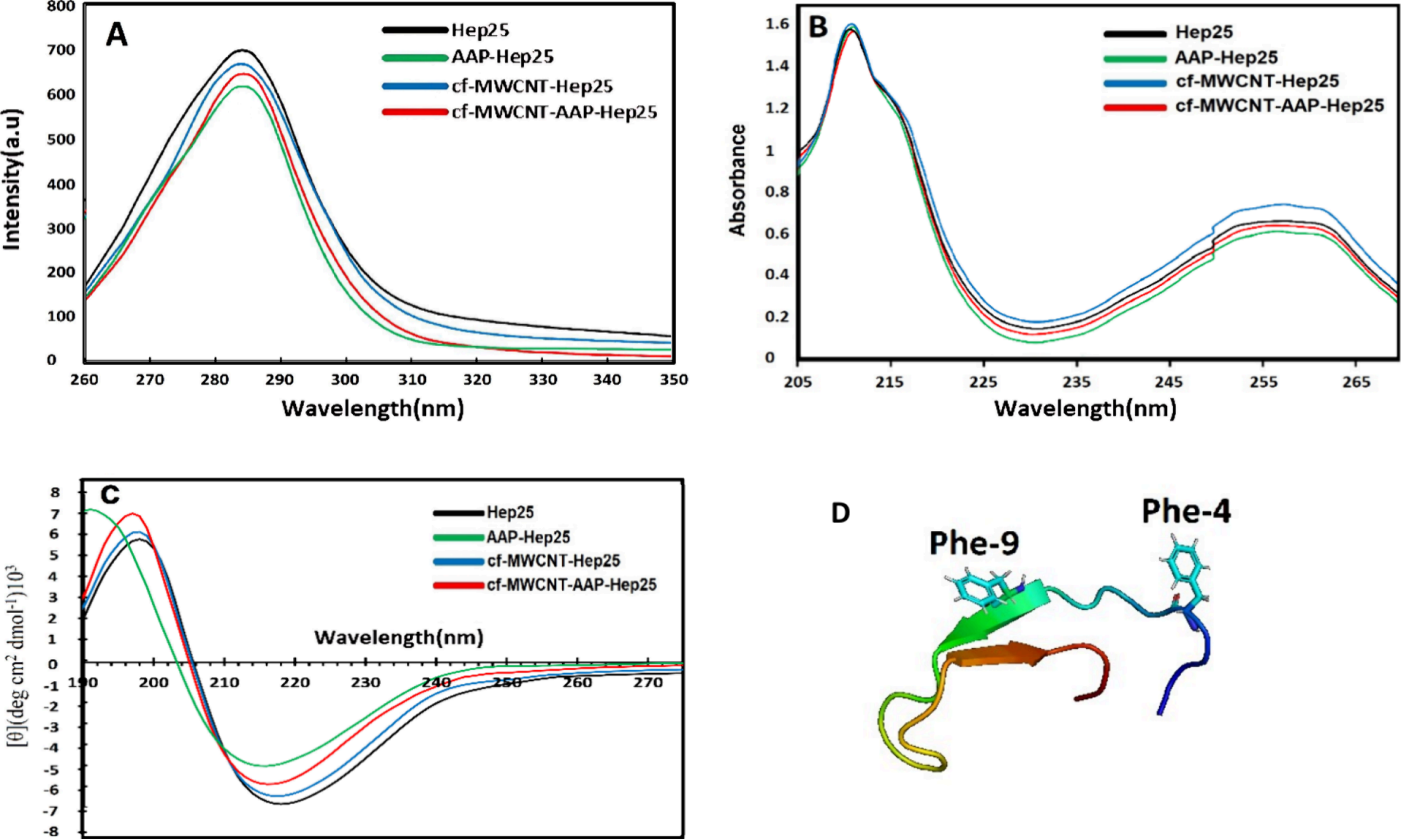
(A) Fluorescence spectra. Conditions: λ_max_ = 280 nm, pH = 7; *C*_Hep25_ = 2.5 × 10^–5^ M. (B) UV–visible absorption spectra (*C*_Hep25_ = 2.5 × 10–5 M) and (C) CD spectra of Hep25 (*C*_Hep25_ = 2.5 × 10^–4^ M), cf-MWCNT-Hep25, cf-MWCNT-AAP-Hep25, and AAP-Hep25. *T* = 298 K, pH 7.0; *C*_CNT_ is in the total volume of the reaction mixture. (D) Position of phenylalanine amino acids in Hep25 blood peptide.

A standard method for investigating ligand–protein interactions is fluorescence, which can provide valuable information about the structure and dynamics of macromolecules such as binding constants, mode of quenching, and binding sites. First, we applied steady-state fluorescence to investigate data on the tertiary structural properties of Hep25 and the binding mechanism of Hep25 with AAP and cf-MWCNT. The intrinsic fluorescence of Hep25 is determined mainly by the phenylalanyl (Phe) residues located within the internal hydrophobic region. Among Hep25 residues, two residues, 4 and 9, are intrinsic fluorophores (Figure 3D). To determine how the conformational properties of Hep25 are affected by cf-MWCNT and AAP, the peptide molecule was allowed to interact with cf-MWCNT and AAP at room temperature, and changes in emission spectra were recorded (Figure 3A). Our experiments measured the fluorescence emission of Hep25 at 282 nm, which is due to the presence of Phe. As shown in Figure 3A, the fluorescence intensity of Hep25 decreases upon the addition of cf-MWCNT and AAP, respectively. Still, the peak intensity for cf-MWCNT-Hep25 was higher in comparison to those for AAP-Hep25 and cf-MWCNT-AAP-Hep25, which could be related to the large surface area of cf-MWCNTs and their ability to adsorb and remove organic molecules with low molecular weights. A slight blue shift can be seen in all complexes, indicating an increase in the hydrophobicity of the peptide surface and confirming the effective conjugation of AAP and cf-MWCNT with Hep25 and the induced conformation changes in Hep25. By introducing cf-MWCNT and AAP to the hydrophobic core, the Phe residues were gradually exposed to the aqueous medium. As a result of the slight blue shift in fluorescence emission spectra, the Phe residues appeared to be in a more hydrophobic microenvironment after the addition of cf-MWCNTs and AAP, suggesting that the cf-MWCNT-Hep25 and AAP-Hep25 interactions are likely to occur around the Phe residues.^48,49^

We used UV–visible absorption to record the effects of cf-MWCNTs and AAP on the Hep25 structure (Figure 3B). The absorption peak of Hep25 at approximately 212 nm resulted from the n → π* electron transition of the aromatic amino acid. In comparison, the UV absorption peak around 257 nm indicates the π–π* of C=O polypeptide bond structures and the energy of the binding orbit in the phenyl groups.^50−52^

As shown in Figure 3B, the absorbance intensity of Hep25 decreased with the addition of AAP, while the absorbance intensity of Hep25 increased after the addition of cf-MWCNTs. The maximum peak position of Hep25 in all samples was red-shifted. At the same time, the combination of cf-MWCNTs and AAP had little effect on the absorption spectra of Hep25. The peak intensity for cf-MWCNT-AAP-Hep25 was slightly higher in comparison to that for AAP-Hep25. The reduced and increased absorbance of AAP-Hep25 and cf-MWCNT-Hep25 suggest Hep25 was combined with phenyl groups within AAP and got adsorbed on the surface of cf-MWCNT, respectively, indicating the relative changes in the side chains of the residues.^53,54^ The findings suggest that the initially hydrophobically shielded backbone structure became exposed to a polar environment.^55^ In essence, the interplay between AAP and Hep25 resulted in the loosening and unfolding of the protein framework.

To further verify the influence of AAP and cf-MWCNTs on the secondary structure of Hep25, CD spectroscopy was performed to monitor rapidly folding and unfolding states and conformational changes^56,57^ (Figure 3C). It has been established that CD spectra in the far-UV region (180–250 nm) provide insights into the secondary structures of proteins, while in the near-UV region (typically spanning 250–350 nm) they offer a means of monitoring the tertiary structures of proteins at the side-chain level.^58,59^ Within the far-UV region, the α-helix and β-sheet secondary structures of proteins exhibit characteristic CD peaks at 192, 208, and 222 nm. In the near-UV region, tertiary structures manifest the following distinctive CD peaks: 255, 261, and 268 nm for Phe; 277 nm for Tyr; and 279, 284, and 291 nm for Trp.^60^ These characteristic CD spectra are valuable for monitoring alterations in a protein’s secondary and tertiary structures. We hence applied the CD spectra (far-UV) of Hep25 in the absence and presence of AAP and cf-MWCNT for the cf-MWCNT-AAP-Hep25 mixture to provide further information about the effects of AAP and cf-MWCNTs on the secondary structure components of Hep25.^61^ The spectral analysis of hepcidin, as depicted in the CD spectrum of Figure 3 C, aligns harmoniously with established findings in the literature.^62^ The spectral profile observed in the CD spectrum indicates a peptide structure featuring disulfide-stabilized β-sheets, exhibiting a defining negative minimum around the 200–220 nm region. The negative peak near 217 nm may facilitate the transfer of electrons from the n → π* orbital of the peptide bond within β-sheets.^63−65^ Any modification in the properties of this decrease might trigger adjustments in the structural integrity of the β-sheets, ultimately impacting their conformational stability and functional behavior. Upon the introduction of AAP to Hep25, there was an evident reduction in the β-sheet composition from 30.49% in the unbound Hep25 to 21.81%, along with a wavelength shift. The diminished proportion of β-sheet content suggests that AAP interacts with the amino acid residues along the backbone chain of Hep25, disrupting key hydrogen bonding patterns and promoting partial protein unfolding. This implies that the binding of AAP to Hep25 initiates significant conformational alterations within Hep25, underscoring the dynamic interplay between the two molecules at a structural level. Besides, the spectral profiles obtained from CD analysis for Hep25, with and without cf-MWCNT, exhibit a discernible resemblance, suggesting a retention of predominant β-sheet secondary structural elements within Hep25 following the interaction with cf-MWCNT. The congruity in CD spectra indicates that the association with cf-MWCNT did not induce significant changes in the dominant secondary structural characteristics of Hep25, underscoring the enduring presence of β-sheet conformations within the peptide configuration. According to the obtained results for the cf-MWCNT-AAP-Hep25 mixture, the binding of cf-MWCNT and AAP to Hep25 could induce some secondary structural changes in Hep25. In addition, the decrease of β-sheet content shows that AAP and cf-MWCNT link to the amino acid residues of the main polypeptide chain of the peptide and distribute their hydrogen bond networks, evidencing that AAP denaturizes Hep25. However, considering that in the structure of Hep25 there is a higher percentage of the β-sheet structure and the structure of the α-helix is less visible, the presence of carbon nanotubes does not change much in the structure of Hep25; as a result, changes in the secondary structure alone are not sufficient to destabilize the spatial structure of Hep25. According to these results, AAP has a more significant effect on hydrogen and disulfide bonds, causing structural changes in Hep25. The findings from the CD analysis are consolidated in Table 1, providing an overview of four secondary structures: α-helix, β-sheet, turns, and disordered.

**Table 1:** Influence of AAP and cf-MWCNT on the Secondary Structure of Hep25

system (experimental)	α-helix (%)	β-sheet (%)	random coil (%)	disordered
Hep25	0.8	30.49	46.72	21.99
AAP-Hep25	0.4	21.81	52.36	25.36
cf-MWCNT-Hep25	0.6	27.43	48.17	23.8
cf-MWCNT-AAP-Hep25	0.5	28.29	48.97	22.24

### 3.2 Computational Section

To delineate the specific binding sites on Hep25, a docking code was employed to simulate the precise binding mode between Hep25 and AAP. Molecular docking investigations involving the Hep25 protein and AAP were undertaken using AutoDock software version 4.2.^33^ The most optimal conformation of the Hep25 peptide, designated by the identifier 1M4F, was retrieved from the RCSB Protein Data Bank (www.rcsb.org). Following the meticulous preparation of the requisite input files for docking, including those for the macromolecule, ligand, and docking map, the investigation was executed to elucidate the intricate interactions between the ligand and peptide, focusing on modeling their dynamics.

A grid measuring 90 × 90 × 90 Å^3^ along the three coordinate axes was established based on the ligand molecular volumes. Molecular docking was employed to examine the interactions between AAP and the Hep25 peptide. Predicted interaction models involving the loop and N-terminus of Hep25, as obtained through molecular docking, were further analyzed using two-dimensional and three-dimensional ligand binding maps. Notably, the binding map associated with the compound exhibiting the most negative energy was explicitly chosen for a detailed investigation.

Molecular docking simulations were conducted to explore the affinity of pollutants, such as AAP, toward the loop and N-terminal regions of Hep25. In this context, AAP served as a reference ligand to investigate the conformational changes of Hep25. Employing the adopted docking protocol, AAP was accommodated within the loop and N-terminus of the Hep25 peptide, forming bonds with various amino acids.

**Figure 4:**
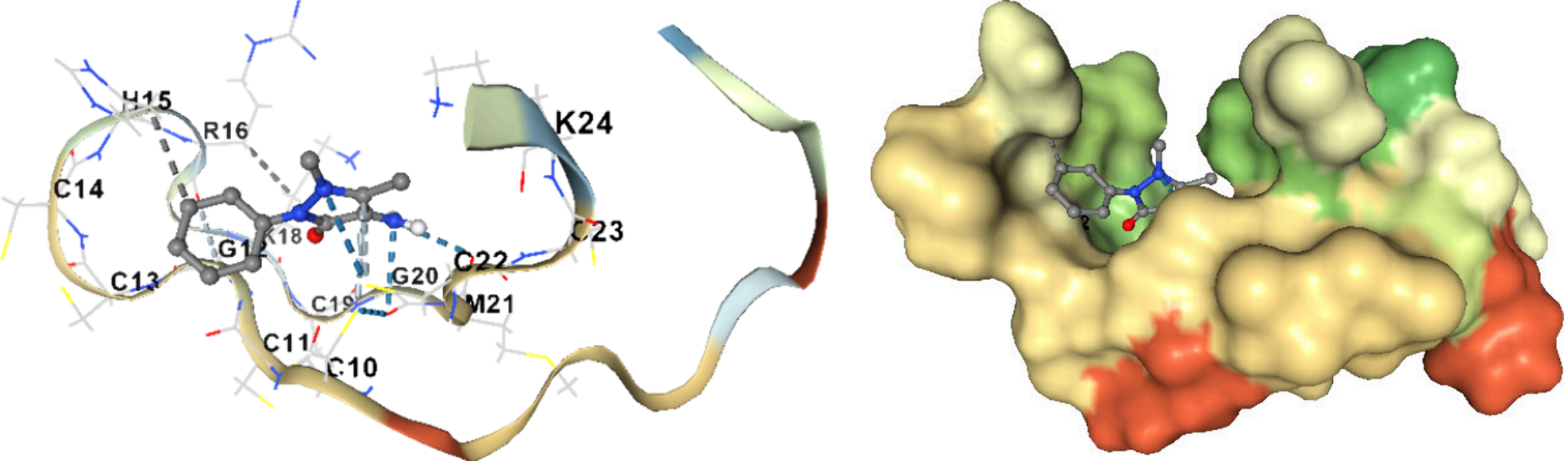
Docking results of the AAP and Hep25 system. (A) Binding site of AAP to Hep25. (B) Detailed illustration of the binding between AAP and Hep25.

A comprehensive molecular docking simulation of the target residues and AAP structure within the Hep25 peptide was executed (Figure 4). Numerous poses were generated, revealing improved binding modes and interactions within the loop and N-terminal regions. Poses exhibiting the most favorable RMSD values, indicative of proximity to the original ligand position within the loop and N-terminus, were selectively identified. As illustrated in Figure 4A, AAP effectively binds within the Hep25 cavity, which is situated amidst the subdomains of Hep25. The docking results of AAP binding with Hep25 are presented in Figure 4B, with the corresponding distances detailed in Table 2. The amino acid residues constituting these binding sites encompass Asp-1, Thr-2, His-3, and Php-4 of the N-terminal area, along with Gly-12, Cys-13, Cys-14, His-15, Arg-16, Ser-17, Lys-18, and Cys-19 of the loop area with more negative energy (stronger binding affinity). The predominant driving force facilitating AAP binding to these sites is electrostatic interaction, aligning with the discussion above. Docking results indicate a lack of hydrogen bonding between Hep25 and AAP, as well as the presence of van der Waals and hydrophobic interactions, emphasizing the prominent role of electrostatic forces in the AAP-Hep25 binding interaction.

**Table 2:** Residues interaction and binding energies of AAP pollutant in the Loop and N-terminal of Hep25 peptide

structures	N-terminal				loop							
amino acid bond	Asp-1	Thr-2	His-3	Php-4	Gly-12	Cys-13	Cys-14	His-15	Arg-16	Ser-17	Lys-18	Cys-19
distance (nm)	0.45	0.43	0.42	0.43	0.28	0.26	0.26	0.27	0.28	0.29	0.30	0.30
RMSD	1.45				1.47							
Sakcal/mol	–3.89				–4.19							
pollutant	AAP				AAP							

**Figure 5:**
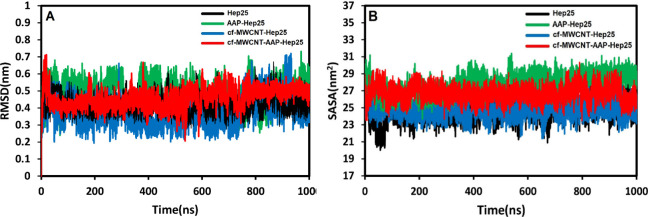
(A) The changes in the RMSD of the Cα atom of Hep25 during the molecular dynamics simulation. (B) SASA of Hep25 in different complexes.

The C_α_ root-mean-square deviation (RMSD) values were calculated over the 1000 ns simulation time to evaluate the structural stability and equilibration of the studied systems. Figure 5A shows the RMSD plot of Hep25 in the presence and absence of the AAP and cf-MWCNTs. As evidenced by the RMSD plots, the Hep25 deviation increased significantly in the presence of cf-MWCNTs and AAP, displaying a rapid divergence from its initial structure. AAP-Hep25 experienced higher fluctuations compared to other systems; as a result, the structure of this complex was observed to be unstable during our MD simulations. Interestingly, the RMSD of the cf-MWCNT-AAP-Hep25 complex indicated a slight difference from the RMSD values of Hep25 before 300 ns. In comparison, more changes in the complex structure were observed after 300 ns. After approximately 780 ns of simulation time, the cf-MWCNT-AAP-Hep25 complex and Hep25 were finally stabilized and preserved their respective structures until the simulation concluded. Hep25 has more fluctuations during the simulation in both mentioned complexes. Furthermore, the average RMSD values of Hep25 and the AAP-Hep25, cf-MWCNT-Hep25, and cf-MWCNT-AAP-Hep25 complexes were approximately 0.42 ± 0.05, 0.48 ± 0.04, 0.37 ± 0.09, and 0.44 ± 0.06 nm, respectively. Consequently, the increasing fluctuations during the simulation follow cf-MWCNT-Hep25 < Hep25 < cf-MWCNT-AAP-Hep25 < AAP-Hep25. Hence, the lower fluctuations of the cf-MWCNT-AAP-Hep25 complex show that the nanotube facilitated the adsorption of the pollutant. This observation suggests that the presence of the organic molecules led to more fluctuations in the system during the simulation. The high degree of deviation in the peptides’ free and complex states will be addressed more thoroughly in the following sections of this work.

It has been shown that hydrophobicity is the primary factor influencing protein folding, while protein misfolding can result in a wide range of diseases, such as blood diseases.^66^ The solvent-accessible surface area (SASA) of biomolecules is often used to assess changes in the hydrophobicity and hydrophilicity of proteins before and after binding to small molecules.^67^ Typically, the solvent accessible surface area is determined as a center of a spherical “solvent” molecule with a radius of 1.4 Å. The surface tension imposed by solvents near their interfaces with proteins affects the protein structure and dynamics.

Figure 5B presents the time evolution graphs of SASAs to solvents of over 1000 ns for all four simulations. In most cases, Hep25 is hydrophobic and composed of β-sheets. The reported results in Figure 5B show that the average SASA values for the complex systems were slightly greater than those of free Hep25 after the addition of the ligands, particularly the AAP-Hep25 complex, suggesting a much more accessible surface for interaction with Hep25. It was observed that Hep25 underwent significant structural changes in the first 70 ns. In contrast, in the first 110 ns of simulation, SASA changes for cf-MWCNT-AAP-Hep25 approached the AAP-Hep25 surface values and reached almost stable values. Thus, Hep25 shows much smaller fluctuations in SASA values when it comes into contact with cf-MWCNT compared to AAP. The SASA values of the four systems were approximately in the range of ∼19–26 nm^2^. According to these data, it can be presumed that the combination of AAP and cf-MWCNT with Hep25 impacted the internal microenvironment of Hep25 and increased the hydrophobic interaction. In contrast, it did not show any apparent change in the hydrophilic surface area of the four systems. Based on our analysis and comparative observations, we found that AAP, in complex with Hep25, changes the structural integrity and relative stability of the complex in water.

**Figure 6:**
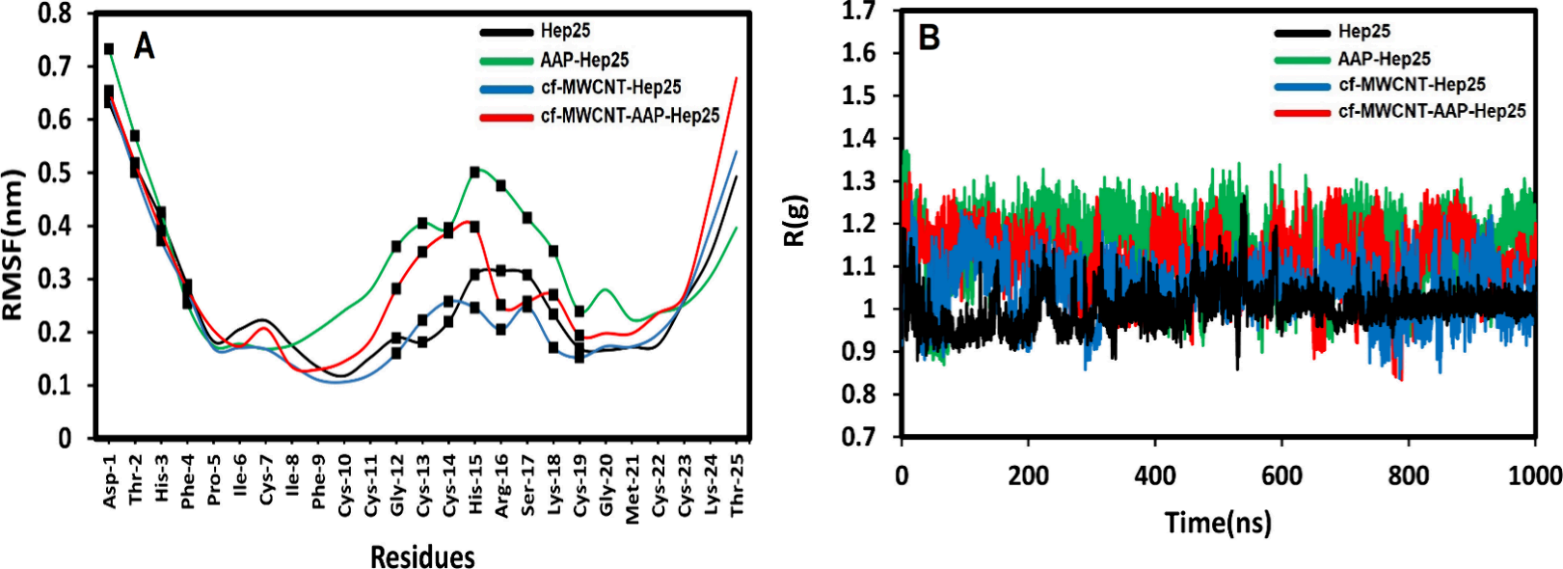
(A) The plot of RMSF for the Cα atom of Hep25. (B) Compactness of the Hep25 backbone according to *R*_g_.

The C_α_ root-mean-square fluctuation (RMSF) was utilized to analyze further deviations in the positions of each protein residue (Figure 6A). In contrast to the RMSD, this analysis provided additional details on how the presence of the ligands affects the flexibility of each peptide residue. It helps detect local changes within a protein chain by using the RMSF method. RMSF plots also show peaks representing residues with the most significant fluctuation. The slight changes in the flexibility of the linker region may impact the interactions between peptides and ligands. The three complexes demonstrated a fluctuation trend of the backbone structure with Hep25. All the complexes showed a change in the RMSF of the C_α_ atom due to amino acid residue participation and interaction. More extensive motions in the region have high RMSF values. According to Figure 6A, the first interesting finding is the high RMSF values in both free and immobilized systems, resulting from the high mobility and flexibility of the four residues located at the initial side chain relating to the N-terminus. The presence of ligands on the side of Hep25 leads to more movement and fluctuations, especially in the central turn regions, showing that the interaction of Hep25 with AAP and cf-MWCNTs has a significant impact on the amino acid residues at the Hep25 binding site, causing changes in the microenvironment surrounding the amino acid residues in Hep25. Additionally, for loop residues, there is an increasing trend in the RMSF values (Hep25 < cf-MWCNT-Hep25 < cf-MWCNT-AAP-Hep25 < AAP-Hep25), indicating obvious evidence of loss of the β-sheets elements. As a whole, the β-sheet regions are more rigid than the unstructured parts of the peptides, oscillating less than the loop regions such as the N-terminal and C-terminal portions of the peptide. Therefore, it is clear that the surface feature of a peptide molecule directly impacts its function when it interacts with others in some ways.

The protein structure’s compactness and stability dynamics were further determined by measuring the radius of gyration (*R*_g_), which displays a folded chain conformation. Hence, the variation in gyration radius over time for all four simulations is shown in Figure 6B.

The *R*_g_ is as follows:
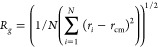
8where *r*_*i*_ and *r*_cm_ represent the position vectors of each atom of the peptide and its center of mass position vector, respectively, and *N* represents the number of atoms in the peptide. According to Figure 6B, the *R*_g_ values of AAP-Hep25 and cf-MWCNT-AAP-Hep25 were greater than those of free Hep25 and cf-MWCNT-Hep25. This may result from the change in the secondary structures of Hep25, such as β-sheet structures caused by the change of microenvironments after binding. As a result, the complexes become relatively loose, so the system expands and loses its original compactness after interacting with cf-MWCNT or AAP. On the other hand, slight variations in average values of *R*_g_ between Hep25 and cf-MWCNT-Hep25 also illustrate the subtle but distinct effects in the structure and flexibility of Hep25 in cf-MWCNT-Hep25. In addition, the *R*_g_ trajectory for Hep25 reached equilibrium in the first 550 ns and maintained the structural integrity until the end of the simulation. In cf-MWCNT-Hep25, the *R*_g_ trajectory shows continuous fluctuations from the beginning of the simulation in comparison to the native structure. It is evident that up to the first 350 s there is a significant loss of structure due to several successive drifts, while after a steady breathing movement up to 750 s the fluctuations continue until the end of the simulation. According to the plot of AAP-Hep25, the AAP exhibits large fluctuations throughout the entire simulation time, indicating the significant loss of residual contacts involved in the regular conformation. In general, the presence of the AAP led to more structural changes in the interaction with Hep25 than cf-MWCNT.

**Figure 7:**
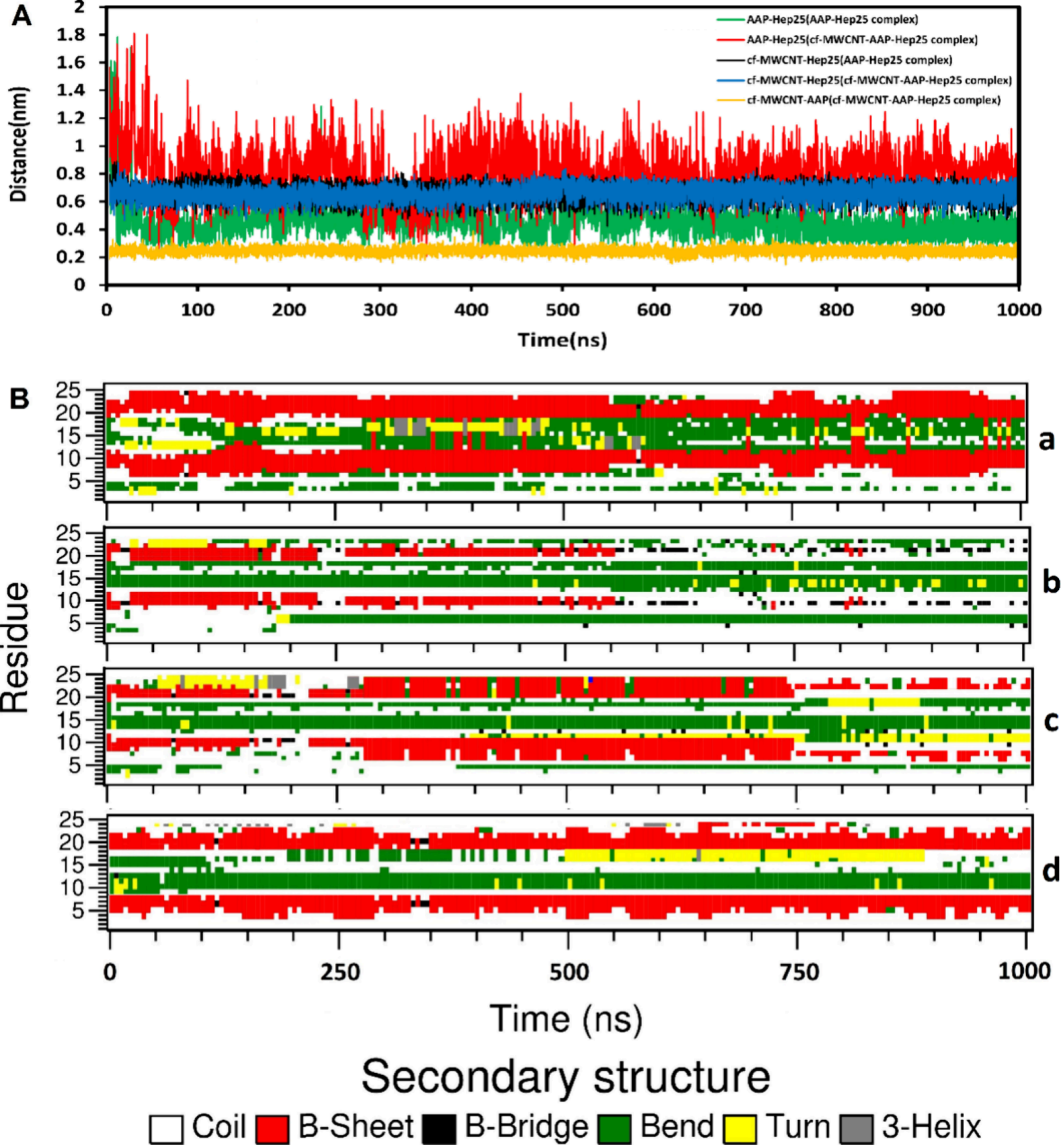
(A)The distance between AAP and Hep25, cf-MWCNT and Hep25, and AAP and cf-MWCNT in AAP-Hep25 and cf-MWCNT-AAP-Hep25 complexes. (B) The secondary structure of Hep25 by DSSP for (a) Hep25, (b) AAP-Hep25, (c) cf-MWCNT-Hep25, and (d) cf-MWCNT-AAP-Hep25 complexes over the duration of the MD simulation.

The center of mass (COM) distances of AAP and Hep25 during the MD simulation in cf-MWCNT-AAP-Hep25 and AAP-Hep25 complexes are shown in Figure 7A. The distance parameter between cf-MWCNT and Hep25 molecules (distance average of 0.69 nm) in cf-MWCNT-Hep25 and cf-MWCNT-AAP-Hep25 complexes has not changed, and the values are very similar. The result means that cf-MWCNT has little effect on Hep25 because Hep25 comprises β-sheet structures and has a high strength. On the other hand, after examining the distance between Hep25 and AAP in Figure 7A, we observed that this value is lower in the AAP-Hep25 complex (distance average of 0.49 nm) than in the cf-MWCNT-AAP-Hep25 complex (distance average of 0.82 nm). On the other hand, the distance between AAP and cf-MWCNT (distance average of 0.29 nm) in the cf-MWCNT-AAP-Hep25 complex is very small, which indicates the interaction of the pollutant with the nanotube. Considering the distance between Hep25 and cf-MWCN as well as the distance between Hep25 and AAP, we notice that AAP moves toward cf-MWCNT, and cf-MWCNT can play an effective role as a pollutant absorber. In addition, it will have the most negligible impact on Hep25 and will cause no interactions between Hep25 and AAP, preserving the structure of Hep25.

To investigate potential changes in the secondary structure of each residue in Hep25, cf-MWCNT-Hep25, AAP-Hep25, and cf-MWCNT-AAP-Hep25, we systematically characterized the Dictionary of Secondary Structure of Proteins (DSSP) throughout the simulations,^68^ as illustrated in Figure 7B. Consistent with previous experimental and theoretical analyses, a substantial portion of the secondary and tertiary structures of peptides tend to be retained during adsorption onto CNTs, with increased vulnerability observed in loop regions. Figure 7B elucidates that, in specific simulations, the peptide’s secondary structure degrades over time from its initial β-sheet structure. Through the computation of the DSSP from the MD simulation, a significant reduction in the β-sheet content of Hep25 was observed upon interaction with AAP and cf-MWCNT. At the same time, the α-helix remained stable throughout the simulation, with minimal alterations. For comparison, the Hep25 structures in the cf-MWCNT-Hep25, AAP-Hep25, and cf-MWCNT-AAP-Hep25 complexes revealed notable distinctions. In the presence of AAP, substantial disruption of the β-sheet portions occurred, leading to the conversion into turn and coil structures. This observation is consistent with our experimental findings and aligns with established trends in the existing literature.^69,70^

Our findings show that the β-sheets are more unstable in the presence of AAP than cf-MWCNT. Hence, it can be asserted that cf-MWCNT does not induce substantial changes in the secondary structure content of Hep25 in comparison to the AAP-Hep25 complex. The findings indicate minimal changes in the greater parts of peptide structures, specifically in the coil and bend regions, following interactions with AAP and cf-MWCNT. In our simulation, loop regions exhibited significant fluctuations in the presence of AAP and cf-MWCNT. Therefore, we focused on the structural changes in the Loop regions. Alterations in β-sheet structures are more prominent in all three complexes, especially in the presence of the AAP. These findings validate that AAP induces substantial changes in the overall conformation of the peptide. Additionally, the absorption sites on cf-MWCNT and electrostatic and π-interactions with AAP contribute to a diminished interaction of AAP with Hep25, resulting in reduced structural changes in the cf-MWCNT-AAP-Hep25 complex. Moreover, sporadic deviations from the β-sheet conformation were identified in the C-terminal, N-terminal, and loop regions of Hep25 within the three complexes. This observation aligns with existing studies, suggesting that a reduction in fluctuation indicates the preservation of the protein structure.

**Figure 8:**
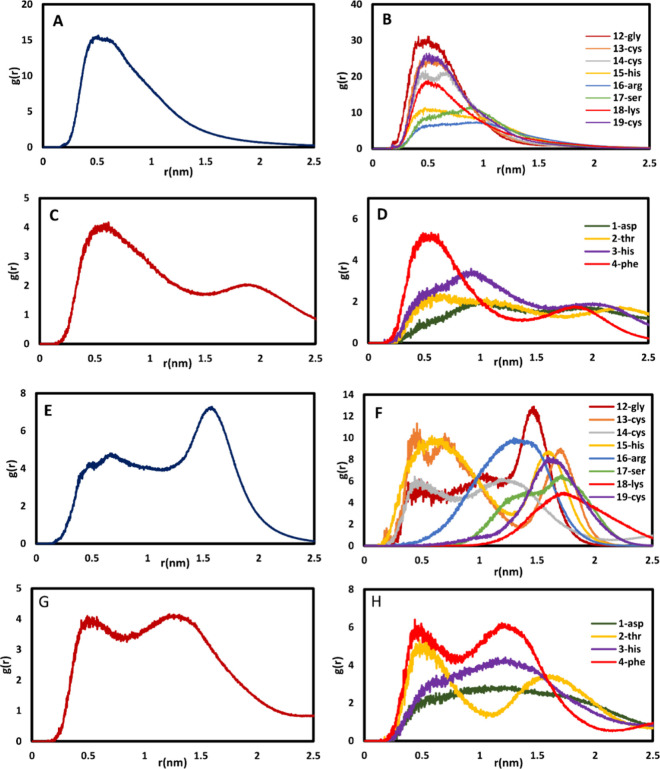
Radial distribution function (RDF) plots. (A) Loop, (b) residues of the loop, (C) N-terminal, and (D) residues of the N-terminal of AAP-Hep25. (E) Loop, (F) residues of the loop, (G) N-terminal, and (H) residues of the N-terminal of cf-MWCNT-AAP-Hep25.

We analyzed radial distribution functions (RDFs) based on data obtained from the MD trajectories of all model systems to evaluate the spatial relationships between amino acids within the proteins and the samples’ surfaces. In this context, RDF quantifies the probability of finding an atom or molecule at a specific distance, denoted as “*r*”, relative to a designated reference atom or molecule. Moreover, examining the RDF between AAp, Hep25, and cf-MWCNT provides significant insights into the structural attributes of cf-MWCNT-AAP-Hep25 conjugates. To elucidate the structural arrangement of the AAP molecule in proximity to the protein, we initiated an assessment of the RDF for the AAP molecule surrounding the Hep25 molecule in all the systems. Figure 8A–D depicts the RDF profiles reflecting the interactions between AAP atoms and the amino acids within the N-terminal and loop peptides.

In contrast, Figure 8E–H illustrates the RDF analysis between the N-terminal and loop peptides in the context of the cf-MWCNT-AAP-Hep25 system. All the relative maxima and minima of the RDFs occur at approximately consistent spatial positions. The maximum values at the lower radius, which are concentrated at shorter distances, are notably higher for the N-terminal structure. However, these curves show a declining trend at extended distances, especially in regions of reduced shorter chain concentration or those devoid of atoms. In Figure 8B and D, it is apparent that the peak intensity of the RDF for Gly-12 in the loop and Phe-4 in the N-terminus of AAP-Hep25 surpasses that of other amino acids, signifying the existence of hydrophobic interactions between the Phe and Gly residues and the AAP acyl groups. Additionally, within the N-terminus of AAP-Hep25, two distinct peaks are evident; the first peak is attributed to the adsorption of amino acids on the AAP surface, while the second peak arises from the elevated molecular density of amino acids within the vicinity of the first peak. For the cf-MWCNT-AAP-Hep25 system (Figure 8E and G), two notable peaks are observed at 0.5 and 1.2 nm. The first peak can be attributed to interfacial van der Waals (vdW) interactions between AAP and the cf-MWCNT surface, while the second peak arises from dense structural arrangements occurring at greater distances from Hep25 following the interaction with cf-MWCNT. A third peak in the loop region might be associated with noninteracting sites along the axial direction of the cf-MWCNT-AAP. Conversely, in the first peak, the peak intensity of the RDF for His-15 and Cys-13 in the loop, along with Phe-4 in the N-terminus, exceeds that of other amino acids (Figure 8F and H). This observation suggests the presence of hydrophilic and hydrophobic interactions with the AAP acyl groups. The results indicate a substantial distribution of the AAP molecule in the proximity of the proteins, spanning approximately 0.50–1.8 nm from the center of mass (COM) of Hep25 at 300 K. This distribution pattern signifies a significant amount of AAP surrounding the protein, displacing water molecules from the protein surface. The strong direct interactions between the AAP molecule and the protein may induce the denaturation of the human peptide. This finding further substantiates our observation regarding the pronounced alignment of amino acids on cf-MWCNT-AAP surfaces, potentially resulting in a higher interaction energy compared to that of the AAP-Hep25 model.

#### 3.2.1 Analysis of Binding Free Energy

**Figure 9:**
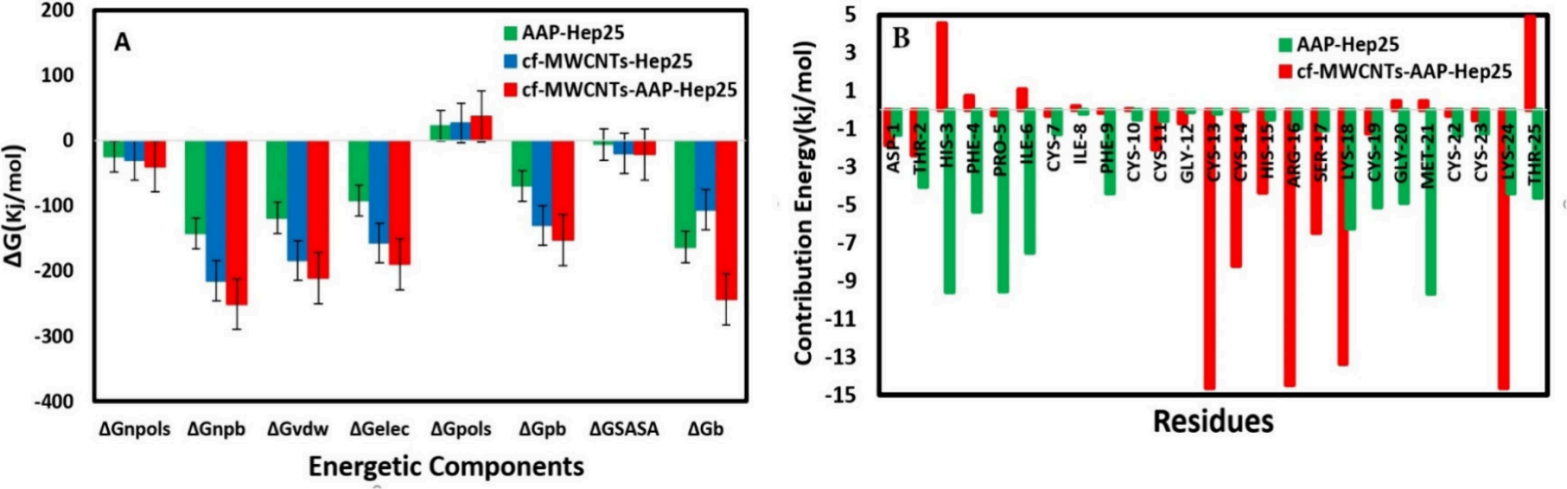
(A) Energetic components of AAP-Hep25, cf-MWCNT-Hep25, and cf-MWCNT-AAP-Hep25. (B) The binding energies (kJ mol^–1^) of residues of AAP-Hep25 and cf-MWCNT-AAP-Hep25 complexes.

To delve deeper into the protein–ligand interactions and ascertain the predominant forces governing binding affinity, we conducted MM/GBSA calculations for binding free energies. Figure 9 illustrates the residues’ average binding free energies and their respective components within the AAP-Hep25, cf-MWCNT-Hep25, and cf-MWCNT-AAP-Hep25 complexes at 300 K.

Figure 9A shows the calculated energies of the selected pollutant on the cf-MWCNT and Hep25 surfaces. As shown, Figure 9A displays negative values for both Δ*G*_vdW_ and Δ*G*_elec_ in the three complexes, signifying the presence of favorable hydrophobic interactions among the complex constituents. This interaction suggests that the adsorption reactions are spontaneous and exothermic. The negative van der Waals energy underscores the robust attraction between AAP, the carbon nanotube, and the proteins. Prior investigations have demonstrated the involvement of π-cation interactions, which entail the interactions between aromatic rings located on the surface of the carbon nanotubes and the cationic groups of basic residues. Additionally, π–π stacking interactions, noncovalent attractive forces between two aromatic rings, have been identified as contributing factors to the binding of carbon nanotubes with proteins.^71,72,70^ Notably, the Hep25 peptides tend to be predominantly enveloped by the hydrophobic tails of the AAP molecule. This outcome aligns with the observation of fewer atoms being adsorbed and a reduced contact area in an aqueous solution. Conversely, the electrostatic solvation energy exhibited a notably unfavorable nature regarding complex formation, reflected in the positive value of Δ*G*_pols_. As previously discussed, the principal driving force behind the binding of AAP with Hep25 is the hydrophobic interaction, consistent with the experimental and docking findings. The negative Δ*G*_b_ values presented in Figure 9A underscore the spontaneity of Hep25 adsorption on cf-MWCNT and AAP. The computed energy values for the cf-MWCNT-AAP-Hep25 complex are notably higher in absolute magnitude compared to those in both cf-MWCNT-Hep25 and AAP-Hep25 complexes, underscoring the presence of robust hydrophobic and π–π interactions between AAP and Hep25. To gain deeper insights into the critical residues contributing to the adsorption process, we conducted a decomposition of the binding energy for each peptide residue concerning both the cf-MWCNT-AAP and AAP surfaces.

The aim was to discern the contribution of specific structural elements, as illustrated in Figure 9B. Figure 9B presents a detailed breakdown of the energy contributions. In the three complexes, virtually all amino acid residues exhibited substantial adsorption on the AAP and cf-MWCNT-AAP surfaces. In the AAP-Hep25 system, 11 residues (His-3, Phe-4, Pro-5, Ile-6, Phe-9, Lys-18, Cys-19, Gly-20, Met-21, Lys-24, and Thr-25) were identified as critical in the adsorption process. For the cf-MWCNT-AAP-Hep25 system, eight residues (Cys-13, Cys-14, His-15, Arg-16, Ser-17, Lys-18, and Lys-24) were discerned as pivotal hot spots in the adsorption process. Moreover, it is evident that several amino acids exhibit substantial adsorption and exert robust interactions within the N-terminus of the peptide in the AAP-Hep25 complex, while in the cf-MWCNT-AAP-Hep25 system such interactions are primarily concentrated within the central loop of the peptide. Among the sampled residues, His-3, Pro-5, and Met-21 in the AAP-Hep25 system (−9.57, −9.55, and −9.67 kcal mol^–1^, respectively) and Cys-13, Arg-16, and Lys-24 in the cf-MWCNT-AAP-Hep25 system (−14.62, −14.45, and −14.63 kcal mol^–1^) stand out as the most significant contributors to the adsorption process. These amino acids consistently displayed the highest affinities in both interaction scenarios. Furthermore, the guanidinium group of Arg-16, the amine groups of Lys-18, Lys-24, Cys-13, Cys-14, and Ser-17 (in the MWCNT-AAP-Hep25 complex), and ILE-6, Lys-18, Lys-24, Cys-19, Gly-20, Met-21, and Thr-25 (in the AAP-Hep25 complex) can engage in multiple interactions, such as π-cation interactions and hydrogen bonds, with cf-MWCNT and AAP.^73^ Additionally, the aromatic residues His-15 (in the cf-MWCNT-AAP-Hep25 complex) and His-3, Phe-4, Pro-5, Phe-9, and Thr-25 (in the AAP-Hep25 complex) can establish interactions with the aromatic rings of cf-MWCNT and AAP through π–π stacking interactions (Figure 9B). As per the MMPBSA results, it is noteworthy that not only negatively charged amino acids (aspartate in the N-terminal) and positively charged residues (histidine, lysine, and arginine in the loop) but also hydrophobic amino acids (glycine, proline, phenylalanine, methionine, and isoleucine) were prominently engaged in contacts with AAP. The unexpected interaction of hydrophobic residues yielded surprising results. Further investigation unveiled two primary reasons for this occurrence. First, certain residues were found close to charged or polar residues, establishing interactions with AAP due to their unavoidable proximity. Second, upon closer examination of the energy analysis, it was discovered that hydrophobic residues made contact with AAP through their backbones. In summary, the interactions of proline, phenylalanine, isoleucine, glycine, and methionine (hydrophobic residues) were inevitable due to their association with AAP, supported by both spatial positioning and energetic considerations. Thus, despite the higher quantity of residues forming van der Waals interactions compared to those forming electrostatic interactions, the latter exhibit lower energy values, rendering them more favorable. This discrepancy arises from the fact that residues forming van der Waals interactions are more widely distributed in the interacting subdomains of each position.

In the AAP-Hep25 complex, the hydrophobic residues of Hep25 play a pivotal role in interactions with AAP. They establish contact with the AAP surface through the aliphatic part (−CH_2_−) and engage in π–π stacking interactions, particularly those involving aromatic residues. In contrast, the polar residues of Hep25 engage in electrostatic interactions with the cf-MWCNT-AAP surface. Generally, aliphatic residues are critical contributors to peptide adsorption on both the AAP and cf-MWCNT-AAP surfaces mediated by van der Waals (vdW) and electrostatic interactions.

An intriguing observation is that the Asp-1 and Thr-25 residues in the cf-MWCNT-AAP-Hep25 system exhibit positive Δ*G*_b_ values, indicating a lack of affinity between these residues and cf-MWCNT-AAP. Therefore, the findings emphasize that Hep25 adsorbs onto the AAP and cf-MWCNT-AAP surfaces through electrostatic interactions, with hydrogen bonds and π–π stacking with the loop region playing a pivotal role in peptide adsorption on the surface of AAP and cf-MWCNTs.^74^ It is pertinent to highlight that a combination of diverse interaction types influences the interaction between Hep25 and AAP or cf-MWCNTs.

## 4 Conclusion

In summary, the interactions of AAP and cf-MWCNTs with Hep25 were examined using multiple spectroscopic methods, including fluorescence, UV–vis absorption, and CD spectra, combined with 1000 ns computational simulations under physiological conditions. The study extensively explored the precise AAP binding site on Hep25 and its impact on the microenvironment and conformation of Hep25 through a comprehensive analysis employing molecular dynamics simulations and molecular docking techniques. Based on the results of fluorescence, UV–vis absorption, and CD spectra, the microenvironment and conformation of Hep25 were demonstrably changed in the presence of AAP. AAP can spontaneously interact with Hep25 through van der Waals and hydrophobic interactions. Furthermore, our results highlighted that the presence of carboxylic acid-functionalized multiwalled carbon nanotubes (cf-MWCNTs) could counteract the detrimental influence of contaminants on Hep25 activity while upholding the fundamental structural integrity of Hep25. The CD analysis shows that a decrease in β-sheet content accompanies the addition of AAP and cf-MWCNTs. The fluorescence spectroscopy results indicate that cf-MWCNTs and AAP effectively quenched Hep25 fluorescence through a static quenching mechanism. This quenching is related to the strong influence of cf-MWCNTs and AAP on Hep25 and the formation of a cf-MWCNT-AAP-Hep25 complex. MD simulations and binding free energy calculations were conducted to ensure the accuracy of our experimental results. Thermodynamic analysis and binding free energy demonstrated that van der Waals forces, π-cation interactions, and π–π stacking are the three dominant forces for the formation of the cf-MWCNT-AAP-Hep25 complex, and AAP and cf-MWCNTs can be spontaneously bound to Hep25. Furthermore, cf-MWCNTs with Hep25 showed fewer structural changes, while AAP made more changes in the Hep25 structure. According to the distance and RDF analysis, the simulation revealed that the introduction of the nanotube prompts the AAP to relocate away from the protein. Consequently, this relocation leads to fewer and weaker interactions between the AAP and the protein. In all simulations, the Cys22 residues were sensitive to ligand adsorption, and significant differences were observed in the loops.

In summary, the MD simulations and experimental data confirmed that the effect of cf-MWCNTs on the unfolding of Hep25 was lower than that of AAP, so the conformation changes, dynamics, and stability of Hep25 are influenced mainly by AAP. In this way, our research can contribute to expanding the use of carbon nanoparticles in environmental pollution cleanup and provide valuable insight into the toxicity mechanism of AAP in association with Hep25. Finally, the findings obtained from the study of the action characteristics of Hep25, cf-MWCNTs, and AAP can be used to disclose the possible mechanism of AAP damage in organisms.
